# FGFR2 Controls Growth, Adhesion and Migration of Nontumorigenic Human Mammary Epithelial Cells by Regulation of Integrin β1 Degradation

**DOI:** 10.1007/s10911-023-09537-x

**Published:** 2023-05-16

**Authors:** Kamil Mieczkowski, Marta Popeda, Dagmara Lesniak, Rafal Sadej, Kamila Kitowska

**Affiliations:** 1 Department of Molecular Enzymology and Oncology, Intercollegiate Faculty of Biotechnology, University of Gdansk and Medical University of Gdansk, Gdansk, Poland; 2 Laboratory Genes and Disease, Department of Dermatology, Medical University of Vienna, Vienna, Austria; 3 Laboratory of Translational Oncology, Intercollegiate Faculty of Biotechnology, Medical University of Gdansk, Gdansk, Poland; 4 Department of Pathomorphology, Medical University of Gdansk, Gdansk, Poland

**Keywords:** FGFR2, integrin β1, mammary gland, epithelial cells

## Abstract

The role of fibroblast growth factor receptor 2 (FGFR2), an important mediator of stromal paracrine and autocrine signals, in mammary gland morphogenesis and breast cancer has been extensively studied over the last years. However, the function of FGFR2 signalling in the initiation of mammary epithelial oncogenic transformation remains elusive. Here, FGFR2-dependent behaviour of nontumorigenic model of mammary epithelial cells was studied. In vitro analyses demonstrated that FGFR2 regulates epithelial cell communication with extracellular matrix (ECM) proteins. Silencing of FGFR2 significantly changed the phenotype of cell colonies in three-dimensional cultures, decreased integrins α2, α5 and β1 protein levels and affected integrin-driven processes, such as cell adhesion and migration. More detailed analysis revealed the FGFR2 knock-down-induced proteasomal degradation of integrin β1. Analysis of RNA-seq databases showed significantly decreased *FGFR2* and *ITGB1* mRNA levels in breast tumour samples, when compared to non-transformed tissues. Additionally, high risk healthy individuals were found to have disrupted correlation profiles of genes associated with FGFR2 and integrin signalling, cell adhesion/migration and ECM remodelling. Taken together, our results strongly suggest that FGFR2 loss with concomitant integrin β1 degradation is responsible for deregulation of epithelial cell-ECM interactions and this process may play an important role in the initiation of mammary gland epithelial tumorigenesis.

**Supplementary Information:**

The online version contains supplementary material available at 10.1007/s10911-023-09537-x.

## Introduction

Morphogenesis of mammary gland is a complex process in which microenvironment of the tissue, i.e. extracellular matrix (ECM) proteins, growth factors, cytokines and hormones, actively interacts with the cellular component of the gland to strictly control formation and further branching of organized structures – milk ducts and acini [1, 2]. Deregulation of communication between epithelial cells and the tissue stroma as well as altered organization of ECM lead to disruption of cell junctions, impaired adhesion to ECM proteins and uncontrolled cell proliferation [3, 4]. All these changes result in disorders of epithelial cell polarity, followed by a dedifferentiation process in the mammary gland, observed already at the early steps of neoplastic transformation [5, 6].

Fibroblast growth factor receptor 2 (FGFR2), as a mediator of signals originating from the tissue microenvironment, has been demonstrated to govern different stages of mammary epithelial morphogenesis [7]. In vivo mouse studies using conditional knock-out of *Fgfr2* revealed its importance in branching and terminal end bud (TEB) formation during early stages of mammary gland development [8]. Among several FGFR2-specific ligands produced during puberty, FGF10 and FGF2 have been identified to play key roles in these processes [9–11]. Moreover, FGFRs have been found to interact with and regulate integrins, the major adhesion receptors for ECM proteins responsible for controlling mammary epithelial cell proliferation and differentiation [12–14]. In particular, Mori et al. proposed a model of the ternary complex formation between FGF1-FGFR1 and integrin αvβ3 [15]. In osteoblasts, activation of FGFR2 was proved to induce integrin α5 ubiquitination and reduced cell attachment to fibronectin [16]. However, to date little is known about the interplay between FGFR2 and integrins in the context of mammary gland biology and pathophysiology. In our previous studies we have demonstrated that in human mammary epithelial cells activated FGFR2 interacts with RSK2, a well-described regulator of integrin function [17], and enhances cell motility [18, 19], suggesting a potential functional cross-talk between FGFR2 and integrins.

In addition to undisputed physiological functions, FGFR2 can also act as an oncogenic factor. Several genome-wide meta-analyses show that single nucleotide polymorphisms (SNPs) in *FGFR2* are strongly associated with an increased risk of breast cancer (BCa) [20–22]. On the other hand, more recent studies demonstrated a positive association between FGFR2 and good prognosis in luminal A BCa patients, highlighting a context-dependent clinical significance of the receptor [23, 24]. However, while the importance of FGFR2 signalling in the context of mammary gland development and BCa progression has been extensively studied, its role in the initial steps of tumorigenesis has not been elucidated.

In this study, we found that FGFR2 knock-down in HB2 cells, a nontumorigenic mammary epithelial cell line in vitro [25] and in vivo model of Ductal Carcinoma In Situ (DCIS) [26], affected morphology of the colonies in three-dimensional (3D) cultures in vitro and downregulated expression of integrins α2, α5 and β1. Furthermore, FGFR2-negative cells exhibited significantly decreased adhesion and migration capabilities towards Collagen type I, Matrigel and Fibronectin. Molecular analyses revealed FGFR2-dependent degradation of integrin β1 – the most prominent integrin receptor. Additional *in silico* analyses comparing breast cancer and adjacent normal tissue showed significantly downregulated expression of *FGFR2* and *ITGB1* in tumour samples. These results suggest that FGFR2 may act as an important factor in the initiation of mammary gland oncogenic transformation.

## Materials and Methods

### Antibodies and Reagents

The following antibodies were used in this study: β-actin (Sigma-Aldrich, clone AC-74), FGFR1 (Santa Cruz Biotechnology, sc-121), FGFR2 (Cell Signaling, #23328), integrin β1 (Cell Signaling, #4706; Santa Cruz Biotechnology, sc-9970), integrin α1 (Santa Cruz Biotechnology, sc-10728), integrin α2 (Santa Cruz Biotechnology, sc-9089), integrin α3 (Santa Cruz Biotechnology, sc-6592), integrin α5 (Chemicon International, AB1949), integrin α6 (Cell Signaling, #3750), LAMP1 (R&D Systems, MAB4800), FGFR (Tyr653/654) (Cell Signaling, #3471), ERK1/2 (Thr202/Tyr204) (Cell Signaling, #9101), DyLight™ 488-conjugated AffiniPure Goat Anti-Rabbit (Jackson ImmunoResearch), AlexaFluor® 680-conjugated AffiniPure Goat Anti-Rabbit (Jackson ImmunoResearch), DyLight™ 594-conjugated AffiniPure Donkey Anti-Mouse (Jackson ImmunoResearch), AlexaFluor® 790-conjugated AffiniPure Donkey Anti-Mouse (Jackson ImmunoResearch), Mouse Anti-Goat IgG-HRP (Santa Cruz Biotechnology, sc-2354). Leupeptin (Sigma-Aldrich) and MG-132 (Selleckchem) were used as inhibitors of lysosomal proteases and proteasome, respectively. AZD4547 (Selleckchem) was used as FGFR inhibitor. For 3D cultures, adhesion assay, cell-spreading assay and migration assay the following extracellular matrix proteins were used: growth factors reduced Matrigel® Basement Membrane Matrix (Corning), rat tail Collagen type I (Millipore) and Fibronectin (Bio-Rad).

### Cell Culture

HB2 cells were purchased from ECACC, grown in DMEM (Corning) supplemented with 10% FBS (Biowest), 5 µg/ml insulin, 5 µg/ml hydrocortisone, 100 U/ml penicillin and 100 µg/ml streptomycin (HyClone), and passaged for a maximum of 2–3 months after reconstitution. Cells were routinely tested for mycoplasma contamination.

### Knock-Down of FGFR2

HB2 cells with stable knock-down of FGFR2 were established with shRNA-based lentiviral construct as previously described [24, 27]. HB2 FGFR2(-) cells were maintained in a medium supplemented with 0.2 µg/ml puromycin (Sigma-Aldrich). In all experiments, respective cells transfected with empty vector were used as controls. The stability of silencing of FGFR2 was verified by immunoblotting before each set of experiments.

### Western Blotting

Cells grown to 70–80% confluence were scraped in ice-cold PBS and lysed in Laemmli buffer (2x concentrated) supplemented with 2 mM PMSF, 10 µg/ml aprotinin, 10 µg/ml leupeptin, 5 mM EGTA, 1 mM EDTA, 2 mM Na_4_P_2_O_7_, 5 mM NaF and 5 mM Na_3_VO_4_. An equal amount of protein (~ 20 µg) per lane was loaded, resolved in SDS-PAGE and transferred onto a nitrocellulose membrane. The membranes were blocked in 5% skimmed milk and incubated overnight with specific primary antibodies at 4ºC. Secondary antibodies conjugated with AlexaFluor® 790 or AlexaFluor® 680 (from Jackson ImmunoResearch) and Odyssey Clx system or secondary antibodies conjugated with horseradish peroxidase (Sigma-Aldrich) and Western Lightning Plus-ECL (PerkinElmer) were used for visualisation of detected proteins. Densitometry of bands representing detected proteins was done with Image StudioTM Software Ver 5.2 (Odyssey CLx).

### Three Dimensional Cell Cultures

1 × 10^3^ HB2 or HB2 FGFR2(-) cells were resuspended in 40 µl (1:1 ratio) of growth factor reduced Matrigel® Basement Membrane Matrix or 0.8 mg/ml of rat tail Collagen type I and cultured in an embedded culture for 8 days. For morphological analyses representative pictures were taken using ZEISS PrimoVert microscope.

### MTT Proliferation Assay

HB2 and HB2 FGFR2(-) cells were seeded into 96-well plate in triplicates. After 72 h the 3-(4,5-dimethylthiazol-2-yl)-2,5-diphenyltetrazolium bromide (MTT, Sigma-Aldrich) was added into each well (0.5 mg/ml) and incubated for 2 h at 37ºC. Then the medium was discarded and formazan crystals were dissolved in DMSO for 15 min at room temperature (RT). The absorbance was measured at 590 nm using microplate reader (BioTek).

### Adhesion Assay

HB2 and HB2 FGFR2(-) cells were seeded into fresh 60-mm cell culture dishes the day before an assay. A 96-well plate was coated with 100 µg/ml of freshly prepared Matrigel® Basement Membrane Matrix, rat tail Collagen type I or Fibronectin overnight at 4ºC. Coating with 100 µg/ml of BSA (Carl Roth) was used as a control. Next, cells were detached with enzyme-free cell dissociation buffer EDTA-based (Millipore) and stained with 10 µM 2’,7’-Bis-(2-Carboxyethyl)-5-(and-6)-Carboxyfluorescein, Acetoxymethyl Ester (BCECF, AM; ThermoFisher) for 15 min at RT, protected from light. After washing, cells were resuspended in serum-free medium and seeded into pre-coated wells (5 × 10^4^ cells per well). Cells were allowed to attach for 90 min at 37ºC. After 3–5 washing steps with PBS, fluorescence signal of the attached cells was measured using a microplate reader (excitation wavelength at 439 nm, emission wavelength at 535 nm).

### Cell-Spreading Assay

HB2 and HB2 FGFR2(-) cells were seeded into fresh 60-mm cell culture dishes the day before an assay. Coverslips were coated with 100 µg/ml of freshly prepared Matrigel® Basement Membrane Matrix, rat tail Collagen type I or Fibronectin overnight at 4ºC. Coated coverslips were washed with PBS and blocked with 1 mg/ml BSA for 30 min. Next, cells were detached with enzyme-free cell dissociation buffer EDTA-based, seeded onto coated coverslips in 12-well plate and allowed to attach and spread for 90 min at 37ºC. After washing with PBS, cells were fixed in 4% paraformaldehyde (PFA) at RT, permeabilised with 0.1% Triton X-100 at 4 °C and mounted with Vectashield® HardSet™ Antifade Mounting Medium with Phalloidin (Vector Laboratories). The extent of cell spreading was quantified as an area of phalloidin signal per cell using ImageJ software. Representative images were taken using ZEISS AxioVert fluorescent microscope.

### Migration Assay

Transwell migration assay was performed as previously described [19]. Briefly, HB2 and HB2 FGFR2(-) cells were seeded into 60-mm cell culture dishes the day before an assay. Next, cells were detached with enzyme-free cell dissociation buffer EDTA-based, resuspended in serum-free medium and 2 × 10^5^ cells were placed in the inner compartment of the Boyden chamber inserts (8 μm pores, BD Bioscence), coated with 100 µg/ml of freshly prepared Matrigel® Basement Membrane Matrix, rat tail Collagen type I or Fibronectin. Cells were allowed to migrate towards the complete medium (10% FBS) for 24 h at 37ºC. The following day, non-migrated cells were removed using a cotton swab. Nuclei of migrated cells were stained with Hoechst and porous membranes were mounted onto glass slides for further analyses using ZEISS AxioVert fluorescent microscope. Migrated cells were counted from 20 random fields and representative images were taken.

### Immunofluorescence

2 × 10^4^ HB2 and HB2 FGFR2(-) cells per well of an 8-well Millicell slide (Millipore) were seeded in 400 µl of complete culture medium and incubated for 24 h at 37ºC. Next day, after fixation with 4% PFA for 10 min at RT, permeabilisation with 0.1% Triton X-100 at 4ºC and blocking with blocking buffer (3% BSA, 3% FBS in PBS) for 1 h at RT, cells were incubated with the desired concentrations of primary antibodies diluted in blocking buffer, overnight at 4ºC. Secondary antibodies conjugated with DyLight™ 488 or DyLight™ 594 (from Jackson ImmunoResearch) together with a counterstain for the nucleus with Hoechst were used for the visualisation of desired target proteins using scanning confocal microscope Leica HCS LSI (Leica).

### RNA Isolation, cDNA Synthesis and RT-qPCR

Total RNA was isolated with TriPURE reagent (Roche) according to manufacturer’s protocol. cDNA was synthesized using the Transcriptor cDNA First Strand Synthesis Kit (Roche). For analysis of *ITGB1* gene expression TaqMan probe Hs00559595_m1 was used. TaqMan probes for *ACTB* (Hs99999903_m1) and *GAPDH* (Hs02786624_g1) were used as reference genes. For qPCR reaction, TaqMan Universal PCR Master Mix (Applied Biosystem) was used. Reactions were prepared in duplicates. Each plate contained a set of non-template controls and controls for gDNA contamination. Gene expression was calculated using a modified ΔΔC approach [28].

### *In silico* Analyses

Two independent publicly available datasets (Normal Breast [29] from UCSC Xena functional genomics platform and Breast Invasive Carcinoma from TNMplot.com integrated database) were used to analyse mRNA levels of *FGFR2* and *ITGB1*, as well as profiles of gene expression correlations in low vs. high cancer risk normal breast tissue samples and paired tumour and adjacent normal breast tissues, respectively [29, 30]. Breast cancer risk estimate in UCSC Xena cohort is based on assigned Gail risk scores [31]. Five-year breast cancer risk threshold to distinguish between low and high-risk patients was set at 1.67% (according to [32]).

### Statistical Analyses

All data were presented as relative mean or as the percentage change ± standard deviation (for experiments repeated at least three times) and an unpaired *t*-test was used to compare the differences between two groups (using GraphPad Prism 8.0.1). For RNAseq datasets analyses differences between two groups were presented as median-based log2 fold change (log2FC) and compared using Mann-Whitney U test. The Kendall’s rank correlation coefficients were calculated for correlation analyses. P-values < 0.05 were considered as statistically significant. Data were analysed and visualized using GraphPad Prism 8.0.1.

## Results

### FGFR2 Controls Growth of HB2 Colonies in 3D Cultures and Expression of Integrins

**Fig. 1:**
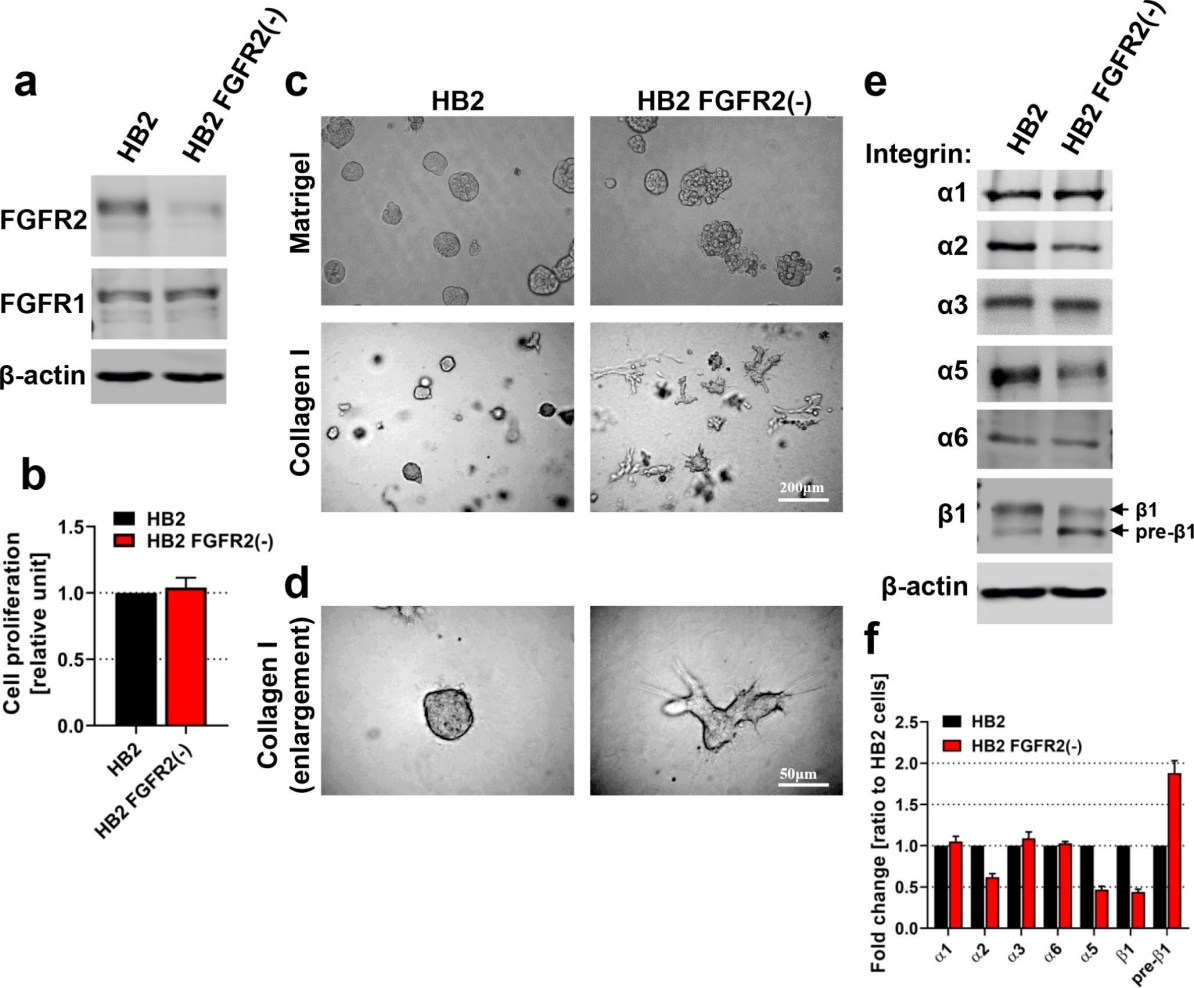
**FGFR2 controls growth of HB2 colonies in 3D cultures and regulates integrins protein level. (a)** The efficiency and specificity of FGFR2 silencing in HB2 cells were confirmed by Western blotting. FGFR3 and FGFR4 were not detectable. **(b)** The effect of FGFR2 silencing on proliferation of HB2 cells was determined by MTT assay. Data are presented as relative means ± SD (n = 3). **(c-d)** HB2 and HB2 FGFR2(-) cells were cultured in 3D Matrigel (C, upper panel) and Collagen type I (c, lower panel; d, higher magnification) for 8 days. Representative images were taken (scale bar: 200 μm and 50 μm, c and d, respectively). **(e)** The effect of FGFR2 silencing on integrins level in HB2 cells was analysed by Western blotting. Arrows indicate mature (upper band) and immature (lower band; pre-β1) integrin β1 forms. **(f)** Densitometry for Western blot analyses of integrins α1, α2, α3, α5, α6 and β1 in HB2 and HB2 FGFR2(-) cells. Data are presented as relative means ± SD (n = 3)

To investigate the importance of FGFR2 in maintaining morphology of human mammary epithelial cells in various ECM environments, first, we established a stable and specific FGFR2 knock-down HB2 cell line variant, here referred to as HB2 FGFR2(-) (Fig. 1a, densitometry in Supplementary Fig. S1a). Noteworthy, silencing of FGFR2 did not affect proliferation rate of HB2 cells (Fig. 1b). As demonstrated in previous studies, HB2 cells formed compact spheric three-dimensional colonies when grown in Matrigel or Collagen type I embedded cultures (Fig. 1c-d, left panel) [25, 26]. However, HB2 FGFR2(-) variant cells exhibited irregular grape-like structures when grown in Matrigel (Fig. 1c, upper right image) or branched ECM-invading structures in Collagen type I (Fig. 1c-d, lower right images). Given that communication between mammary epithelial cells and surrounding ECM proteins during mammary gland development and physiology, as well as breast cancer initiation are driven by integrins [33, 34, 14], we next assessed the effect of FGFR2 knock-down in HB2 cells on the expression level of specific integrins. Western blot analysis showed decreased protein levels of integrin α2, α5 and mature form of β1 (with concomitant deregulation of the ratio between mature and immature precursor form; here referred to as pre-β1) in HB2 FGFR2(-) cells, whereas α1, α3 and α6 remained unchanged (Fig. 1e, densitometry in Fig. 1f), indicating a possible molecular link between FGFR2 activity and mammary epithelial cell-ECM communication involving integrins function. Additionally, treatment of HB2 cells with FGFR inhibitor AZD4547 significantly decreased protein level of integrin β1 after 48 and 72 hours, simultaneously with the most pronounced inhibition of FGFR (here reflected in decrease of FGFR and ERK1/2 phosphorylation) (Supplementary Fig. S1b). These results further corroborated our findings from FGFR2 silencing, however here the ratio between mature and precursor form of integrin β1 remained unchanged.

### FGFR2 Regulates HB2 cell Adhesion and Migration Towards Specific ECM Proteins

**Fig. 2:**
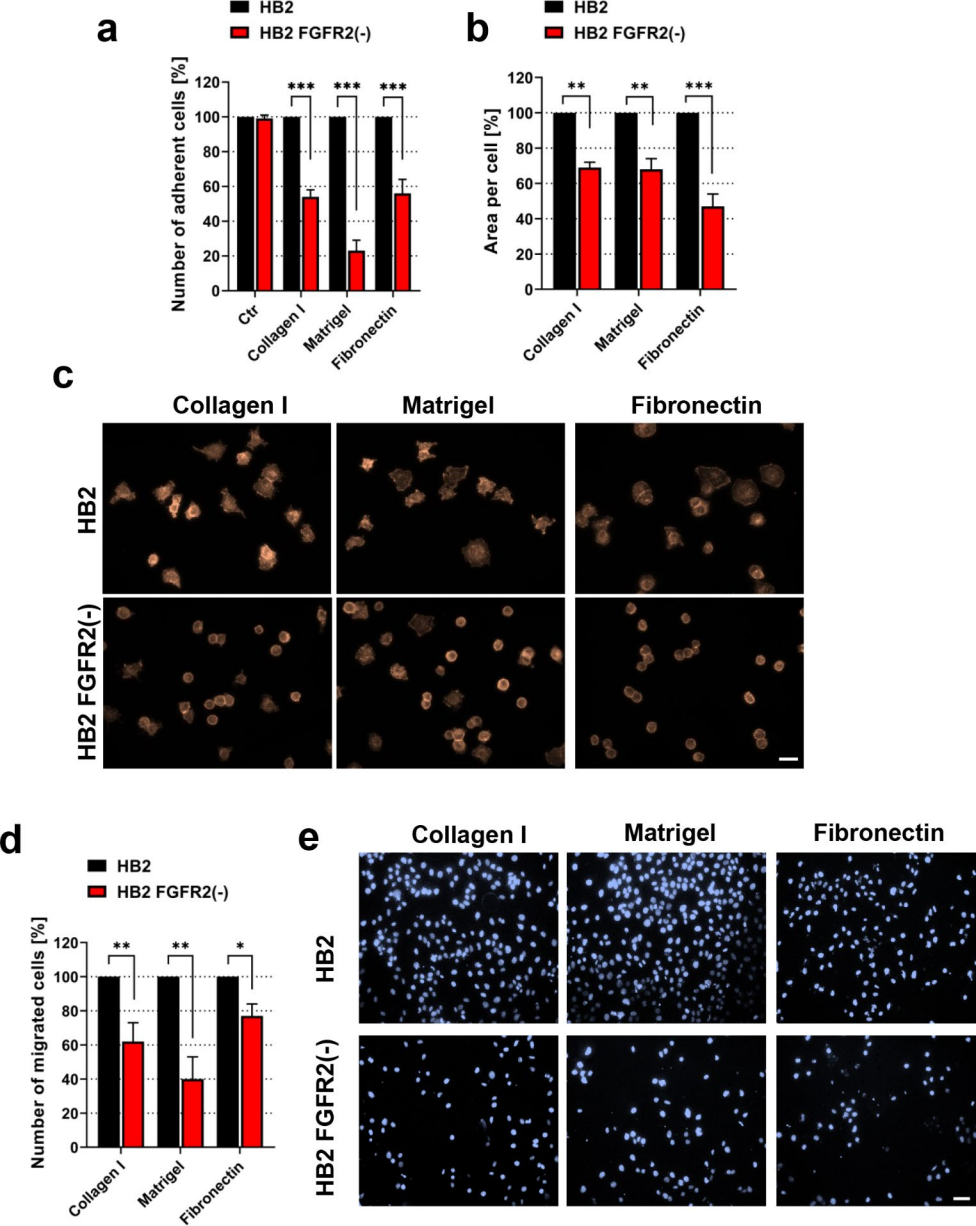
**FGFR2 regulates HB2 cell adhesion and migration towards specific ECM proteins. (a)** The effect of FGFR2 silencing on adhesion of HB2 cells to Collagen type I, Matrigel and Fibronectin was analysed by adhesion assay. Cells were allowed to adhere to wells coated with 100 µg/ml of each ECM protein for 90 min. BSA-coated well (100 µg/ml) was used as a control (Ctr). **(b-c)** Quantitative analysis of HB2 and HB2 FGFR2(-) cells spreading was performed by cell spreading assay. ECM proteins concentration and adhesion/spreading time were the same as for adhesion assay. Actin filaments were stained with TRITC-Phalloidin. Cell areas were quantified by ImageJ software **(b)** and representative images were taken (scale bar: 20 μm) **(c)**. **(d-e)** The effect of FGFR2 silencing on migratory potential of HB2 cells was analysed by Transwell migration assay. Number of migrated cells was counted **(d)** and representative images were taken (scale bar: 40 μm) **(e)**. Cell adhesion, spreading and migration assays data are presented as the percentage change of number of adherent/migrated cells or cell area ± SD (n = 3–5), *p < 0.05, **p < 0.005, ***p < 0.001 by Student’s *t* test

α2β1 and α5β1 integrin heterodimers exhibit the highest affinity towards collagens and RGD motif-containing matrix proteins (e.g. Fibronectin), respectively, and are involved in the process of branching morphogenesis during mammary gland development [13, 35]. On the other hand, integrin β1 subunit forms heterodimers with α1–11 subunits, covering a wide range of interacting ECM proteins. To further functionally explore the role of FGFR2 in integrin-dependent processes, HB2 and HB2 FGFR2(-) cells were subjected to adhesion and migration assays. Adhesion assay results show significantly decreased number of attached HB2 FGFR2(-) cells when compared to wild-type counterpart, with a similar decrease for Collagen type I and Fibronectin (44% and 46%, respectively), and more prominent difference for Matrigel adhesion (76% lower than wild-type cells) (Fig. 2a). Additionally, to analyse adherence to these substrates in a more detailed fashion, cell spreading assay was performed. Quantitative analysis of cell area after 90 min of adhesion shows similar reduction for HB2 FGFR2(-) cells when seeded onto Collagen type I and Matrigel (31% and 32%, respectively), and significantly decreased cell spreading capacity onto Fibronectin (53% of decrease of area per cell) (Fig. 2b). Interestingly, as depicted in Fig. 2c, FGFR2 knock-down in HB2 cells affected not only cell spreading per se, but also the formation of cell protrusions and actin cytoskeleton organization (Fig. 2c). Furthermore, transwell cell migration assay revealed significantly decreased migratory abilities of HB2 FGFR2(-) cells towards all tested substrates in a pattern similar to that observed in adhesion assay (Fig. 2d-e). These results confirm a functional link between FGFR2 and integrin-dependent mammary epithelial cell behaviour.

### FGFR2 Regulates Degradation of Integrin β1

**Fig. 3:**
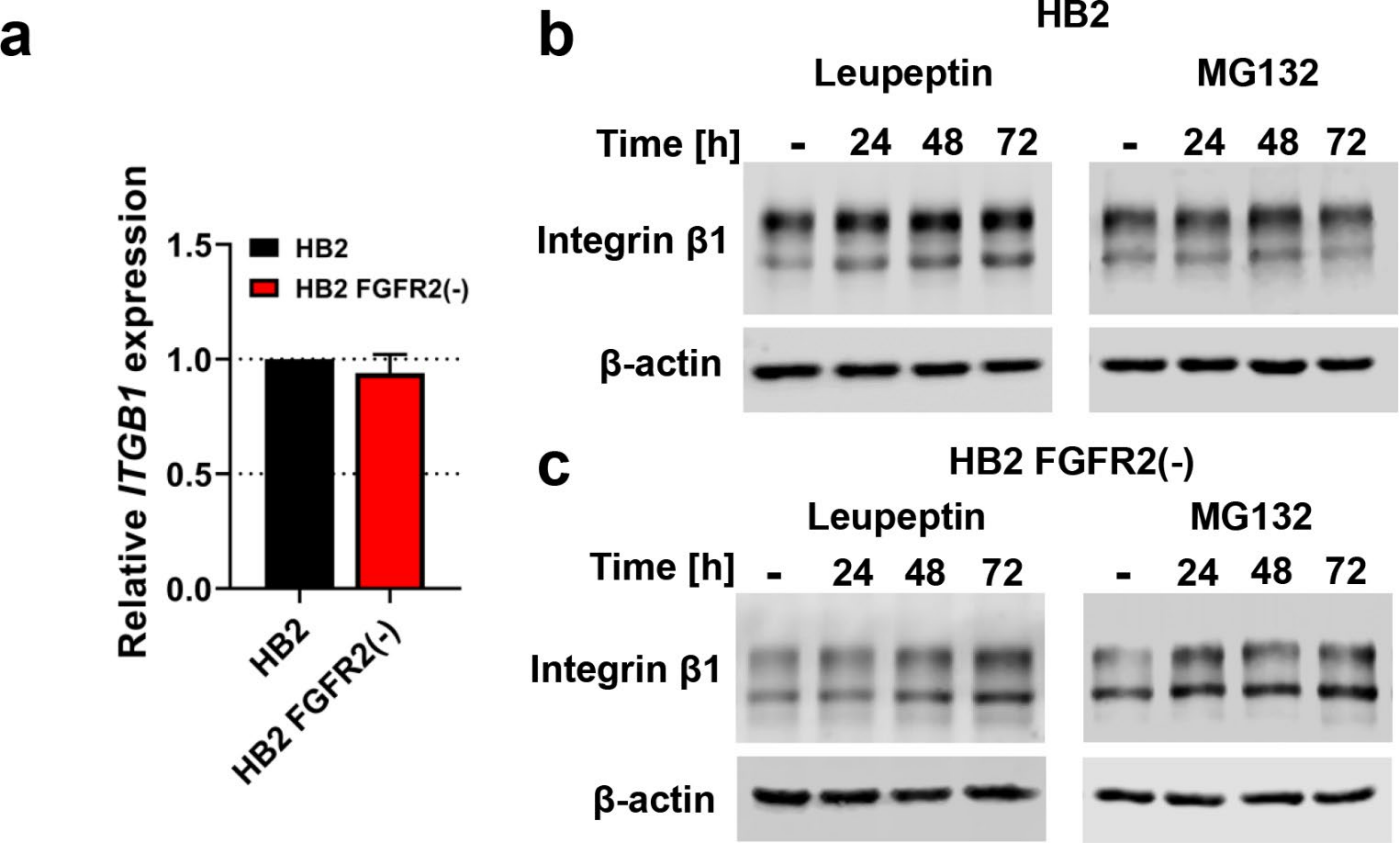
**FGFR2 regulates degradation of integrin β1. (a)** Relative expression of *ITGB1* in HB2 and HB2 FGFR2(-) cells was analysed by RT-qPCR. Data presented as means ± SD (n = 3). **(b)** HB2 cells were incubated with Leupeptin (100 µg/ml) and MG132 (200 nM) for 24, 48 and 72 h. Integrin β1 protein level was analysed by Western blotting. β-actin was used as a protein loading control. **(c)** HB2 FGFR2(-) cells were incubated with Leupeptin (100 µg/ml) and MG132 (200 nM) for 24, 48 and 72 h. Integrin β1 protein level was analysed by Western blotting. β-actin was used as a protein loading control

As previously mentioned, the most conspicuous integrin receptor, integrin β1, interacts with a vast majority of α subunits and is responsible for binding to ECM proteins, regulating cell attachment and migration [36]. Therefore, regulation of integrin β1 function is critical for controlling integrin-dependent interactions with ECM. In the next step of the study, we analysed the possible mechanism of described decrease of the mature form of integrin β1 level in HB2 FGFR2(-) cells. First and foremost, qPCR analysis showed no significant differences in *ITGB1* gene (coding integrin β1) expression between HB2 and HB2 FGFR2(-) cells (Fig. 3a). To verify that observed FGFR2-mediated downregulation of integrin β1 (Fig. 1e) results from protein degradation we used inhibitors of lysosomal and proteasomal pathways - leupeptin and MG132, respectively. Western blot analysis showed that abrogation of canonical lysosomal degradation led to modest, but consistent increase of integrin β1 level in HB2 cells during 24–72 h of incubation with leupeptin (Fig. 3b, left panel; densitometry in Supplementary Fig. S2a). FGFR2 silencing changed the kinetics of an observed increase, reaching the most significant difference at 72 h timepoint (Fig. 3c, left panel; densitometry in Supplementary Fig. S2b). However, while MG132 treatment did not cause any significant effect on integrin β1 level in HB2 cells (Fig. 3b, right panel; densitometry in Supplementary Fig. S2a), it restored and stabilized integrin β1 level throughout the whole incubation period in HB2 FGFR2(-) cells (Fig. 3c, right panel; densitometry in Supplementary Fig. S2b). Noteworthy, an inverted ratio of mature to immature integrin β1 form in HB2 FGFR2(-) cells was observed in all applied treatments (Fig. 3c). Furthermore, immunofluorescent colocalization experiments of integrin β1 and lysosomal-associated membrane protein 1 (LAMP1) showed no differences in colocalization of both proteins between HB2 and HB2 FGFR2(-) cells (Supplementary Fig. S2c), although enhanced accumulation of lysosomes in perinuclear cell region of HB2 FGFR2(-) cells was observed (Supplementary Fig. S2c, arrowheads), indicating potentially enhanced trafficking pathways mediated by FGFR2 knock-down. These results suggest that FGFR2 may impair integrin β1 degradation in the 26 S proteasome complex in HB2 nontumorigenic mammary epithelial cells, however a specific mechanism involved in this protection needs to be further explored.

### Expression Level of *FGFR2* is Decreased in high-risk Normal and Tumour Breast Tissues

**Fig. 4:**
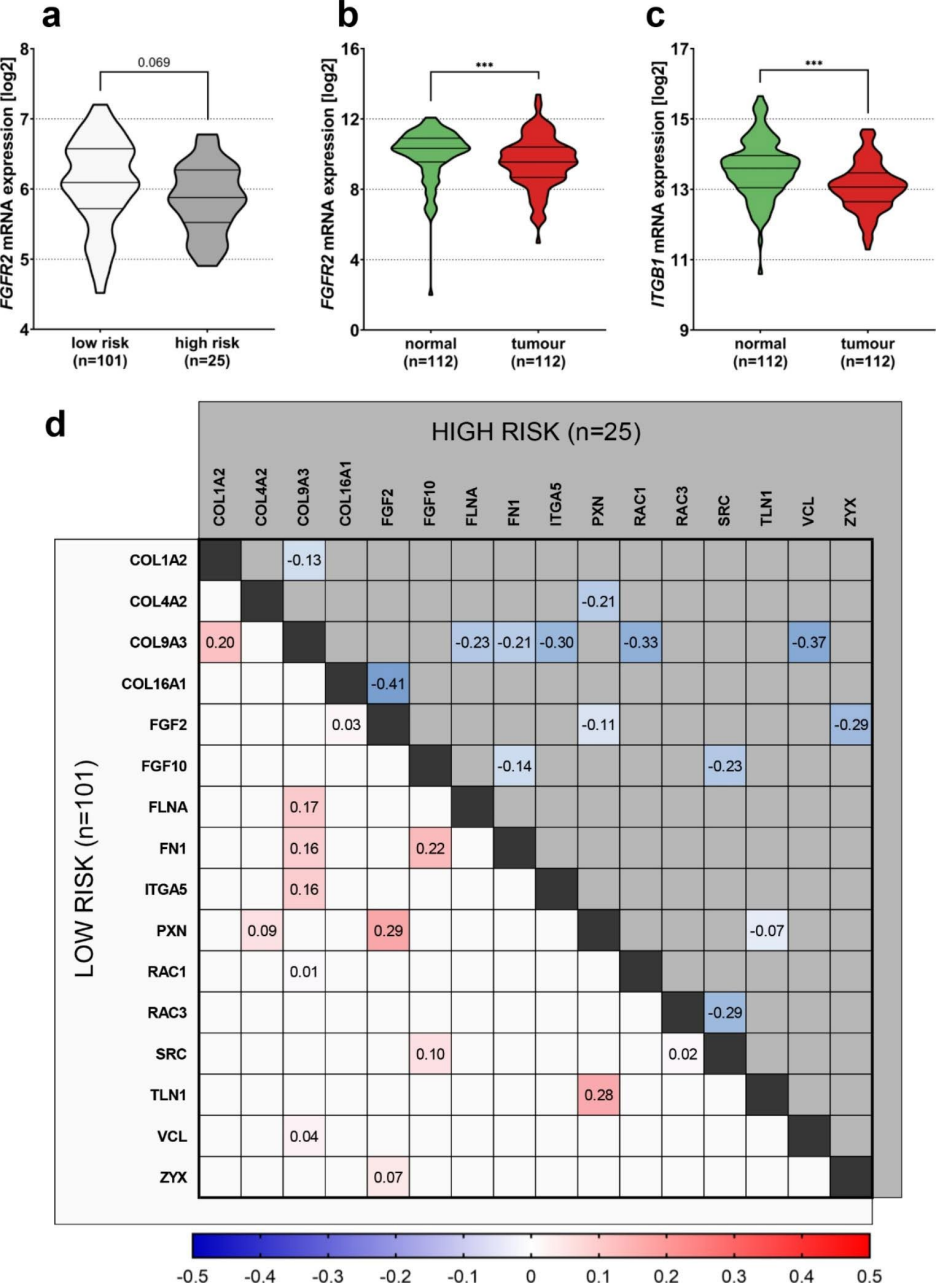
**Expression level of *FGFR2 *is decreased in high-risk normal and tumour breast tissues. (a)** Normal breast expression of *FGFR2* according to breast cancer risk estimate (based on Gail score). p-value was calculated using Mann-Whitney U test. *FGFR2 ***(b)** and *ITGB1 ***(c)** expression in paired breast normal and tumour tissues (n = 112). p-values were calculated using Mann-Whitney U test. **(d)** Correlation matrix of expression of selected cell adhesion/migration, ECM composition and organization, and FGFR signalling-associated genes in low (n = 101, bottom left part of the matrix) and high (n = 25, upper right part of the matrix) risk normal breast samples, with positive correlations shown as red and negative as blue intensity-scaled τ values. Correlation coefficients were calculated using Kendall rank correlation. Only gene pairs with reverse τ sign and absolute τ difference between high and low-risk group higher than 0.3 are depicted

Disruption of the cell-ECM interactions as well as aberrant ECM remodelling may be the first steps in the process of oncogenic transformation [37]. Thus, we evaluated the mRNA expression level of *FGFR2* and *ITGB1* in two independent databases: (i) UCSC Xena functional genomics platform (Normal Breast Benz 2020 dataset), comprising of normal breast samples from 126 healthy women with assigned breast cancer risk estimate (Gail score) [29], and (ii) TNMplot.com web platform (Breast Invasive Carcinoma dataset), enabling to compare normal and tumour paired transcriptomic data [30]. Expression distribution data compared between low (n = 101) and high (n = 25) risk healthy breast samples showed a tendency for *FGFR2* expression to decrease in high-risk patients (log2FC=-0.21, p = 0.069; Fig. 4a). These data were further elaborated by analysis of RNAseq data from paired tumour and adjacent normal tissues (n = 224, including 112 pairs) at TNMplot.com platform. Here, *FGFR2* (Fig. 4b) and *ITGB1* (Fig. 4c) expression levels were both significantly decreased in tumour samples (log2FC=-0.78, p = 0.006, and log2FC=-0.54, p < 0.00001, respectively). Additional correlation analyses on UCSC Xena dataset showed that expression correlation profiles of genes associated with integrin regulation and signalling (*FLNA*, *RAC1*, *RAC3*, *SRC*, *TLN1*), cell adhesion/migration (*ITGA5*, *PXN*, *VCL*, *ZYX*), ECM composition and organization (*COL1A2*, *COL4A2*, *COL9A3*, *COL16A1*, *FN1*), as well as FGFR2 signalling (*FGF2*, *FGF10*) differ between low and high-risk patients, with several gene pairs showing similar, weak-to-moderate positive expression correlations in low-risk patients, which are further disrupted or reverted in high risk patients (Fig. 4d). Importantly, among genes included in the correlation analyses were genes encoding ECM proteins used in the described in vitro part of the study, i.e. Collagen type I, Fibronectin or Collagen type IV (one of the component of Matrigel). Taken together, *in silico* analyses support our in vitro data suggesting that FGFR2 may be an important receptor in non-transformed mammary gland and decrease of its level, concomitant with disruption of cell-ECM communication, might be a feature of oncogenic transformation.

## Discussion

Given that FGFs/FGFR2 signalling is involved in mammary epithelial morphogenesis and ensures normal gland development and physiology [7, 11, 38–40], its expression level and activity need to be tightly controlled. Deregulation of FGFR2-driven signalling may result in impaired ductal branching and disabled development of mammary gland during morphogenesis [8, 10], as well as tumour formation and further progression of the disease in adults [41, 42]. Although many key aspects of FGFR2 signalling in all abovementioned processes have been extensively studied, the role of FGFR2 in the initiation of breast tumorigenesis remains elusive. Our study demonstrates for the first time that reduced FGFR2 expression, with concomitantly decreased integrins levels, affects mammary epithelial cell phenotype in 3D cultures, cell adhesion and migration – characteristic features of oncogenic transformation.

Cross-talk between integrins and growth factor receptors (GFRs), including FGFRs, has been described in several studies [43, 44]. On the one hand, integrins regulate FGFRs signalling by direct binding of some well-known FGFRs ligands, e.g. FGF1 [15], change of the receptors expression level [45] or by convergence of common intracellular signalling pathways [46]. On the other hand, integrins` function can be controlled in two ways: (i) by conformational changes of their extracellular domains (inside-out signalling), and (ii) by regulation of integrins internalisation, intracellular sorting and targeting into degradation [47, 48]. Our previously published data described p90 ribosomal S6 kinase 2 (RSK2) as a binding partner and a downstream effector of FGFR2 in mammary epithelial as well as in BCa cell lines [18, 19], which was additionally shown to regulate integrin activation and promote cell motility [17]. Moreover, FGFR2 activation in osteoblasts was proved to drive Cbl-mediated ubiquitination of α5 subunit and its subsequent degradation in proteasome [16]. On the contrary, our results from this study showed that FGFR2 protects integrin β1 from degradation in 26 S proteasome in nontransformed mammary epithelial cells. Whether it is associated with altered integrin intracellular tail ubiquitination [49] or impaired endocytosis and trafficking [50] remains to be further explored. It should be additionally noted that FGFR2 knock-down cells were characterized by decrease of only mature (a fully active) form of integrin β1, disrupting the ratio between mature and immature precursor form of this integrin. Interestingly, a selective FGFR inhibitor AZD4547 treatment decreased protein level of both forms, indicating a more specific FGFR2-dependent role in this process. Integrin β1 receptor maturation reflects its extensive glycosylation and is associated with the activity of presenilins or alkaline ceramidase 2 [51–53]. It is possible that reduced HB2 FGFR2(-) cell adhesion, spreading and migration towards Collagen type I, Matrigel and Fibronectin were associated not only with increased degradation, but also with impaired maturation of integrin β1.

Results of our *in silico* analyses supported the in vitro investigations, indicating that in high-risk normal breast samples, in contrast to low-risk group, *FGFR2* mRNA levels tended to be decreased. The major limitation of the analysis was a relatively small group of high-risk patients (n = 25). Hence, we employed another database for a similar analysis, with paired invasive BCa patients samples and adjacent nontransformed tissue RNAseq data. This supported previously observed tendency i.e. *FGFR2* mRNA expression was significantly lower in BCa samples with, importantly, a concomitant decrease of *ITGB1* level. It is important to note that no correlation between *FGFR2* and *ITGB1* transcripts has been identified. These results showed once again that the nature of FGFR2 in transition from healthy mammary gland to BCa is not that intuitive. Although hotspot mutations and SNPs within *FGFR2* are being considered as oncogenic [54–57], high-risk normal and invasive BCa samples exhibited decreased *FGFR2* levels. Additionally, correlation analyses of the high- vs. low-risk healthy patients were in line with other previously published data showing that uncontrolled ECM remodelling and reorganization are characteristic for high-risk breast tissue [58, 59]. This is particularly interesting in light of recent studies focused on context-dependent prognostic significance of FGFR2 in invasive BCa [23, 24] and identification of highly oncogenic truncated *FGFR2* variant [60]. Since clinical responses to various FGFR2 inhibitors among BCa patients have still remained highly variable, there is the need to identify and better understand co-regulators of FGFR2-dependent cellular functions at the different stages of disease progression.

Summing up, our functional and molecular analyses combined with *in silico* exploration provided strong evidence that FGFR2 loss may be associated with the early events of mammary gland oncogenic transformation. Described in this work FGFR2/integrin β1 interdependence seems to play a crucial role in the maintenance of cell-ECM communication, a key determinant of healthy mammary gland epithelial cells.

## Electronic Supplementary Material

Below is the link to the electronic supplementary material.

Supplementary Material 1: Fig. S1 (a). Densitometry for Western blot analyses of FGFR1 and FGFR2 in HB2 and HB2 FGFR2(-) cells. **(b)** HB2 cells were incubated with AZD4547 (5 µM) for 24, 48 and 72 h. Integrin β1, FGFR (Tyr653/654) and ERK1/2 (Thr202/Tyr204) protein levels were analysed by Western blotting. β-actin was used as a protein loading control.

Supplementary Material 2: Fig. S2 (a-b). Densitometry for Western blot analysis of integrin β1 mature form (upper band) degradation pathways in HB2 **(a)** and HB2 FGFR2(-) **(b)** cells upon Leupeptin (left panels) and MG132 (right panels) treatment. **(c)** Localization of LAMP1 and integrin β1 in HB2 (upper panel) and HB2 FGFR2(-) (lower panel) cells. Accumulation of LAMP1 stained lysosomes indicated by arrowheads. Representative pictures taken from at least three independent experiments. Scale bar: 10 μm.

## Data Availability

The data that support the findings of this study are available from the corresponding authors upon reasonable request.

## Abbreviations

BCa: Breast cancer
DCIS: Ductal carcinoma in situ
ECM: Extracellular matrix
FGFR2: Fibroblast growth factor receptor 2
LAMP1: Lysosomal-associated membrane protein 1
TEB: Terminal end bud
