# Supplementary figures and images for: FGFR2 Controls Growth, Adhesion and Migration of Nontumorigenic Human Mammary Epithelial Cells by Regulation of Integrin β1 Degradation
Source: J Mammary Gland Biol Neoplasia. 2023 May 16;28(1):9. doi: 10.1007/s10911-023-09537-x (PMC10188388; doi:10.1007/s10911-023-09537-x)

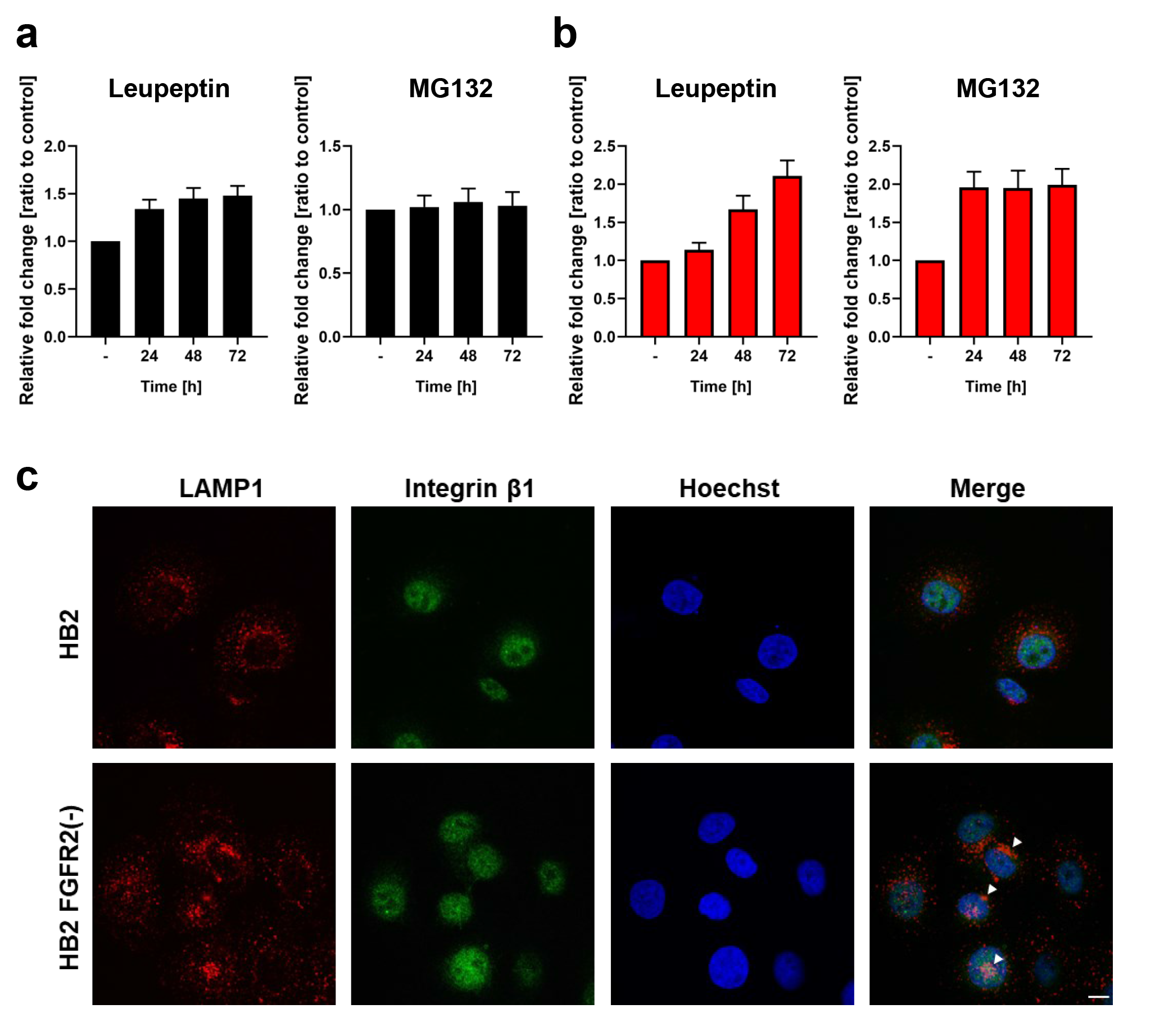

Supplement: Supplementary file 1 — Supplementary Material 1: Fig. S1 (a). Densitometry for Western blot analyses of FGFR1 and FGFR2 in HB2 and HB2 FGFR2(-) cells. (b) HB2 cells were incubated with AZD4547 (5 µM) for 24, 48 and 72 h. Integrin β1, FGFR (Tyr653/654) and ERK1/2 (Thr202/Tyr204) protein levels were analysed by Western blotting. β-actin was used as a protein loading control. [file 10911_2023_9537_MOESM1_ESM.tif]

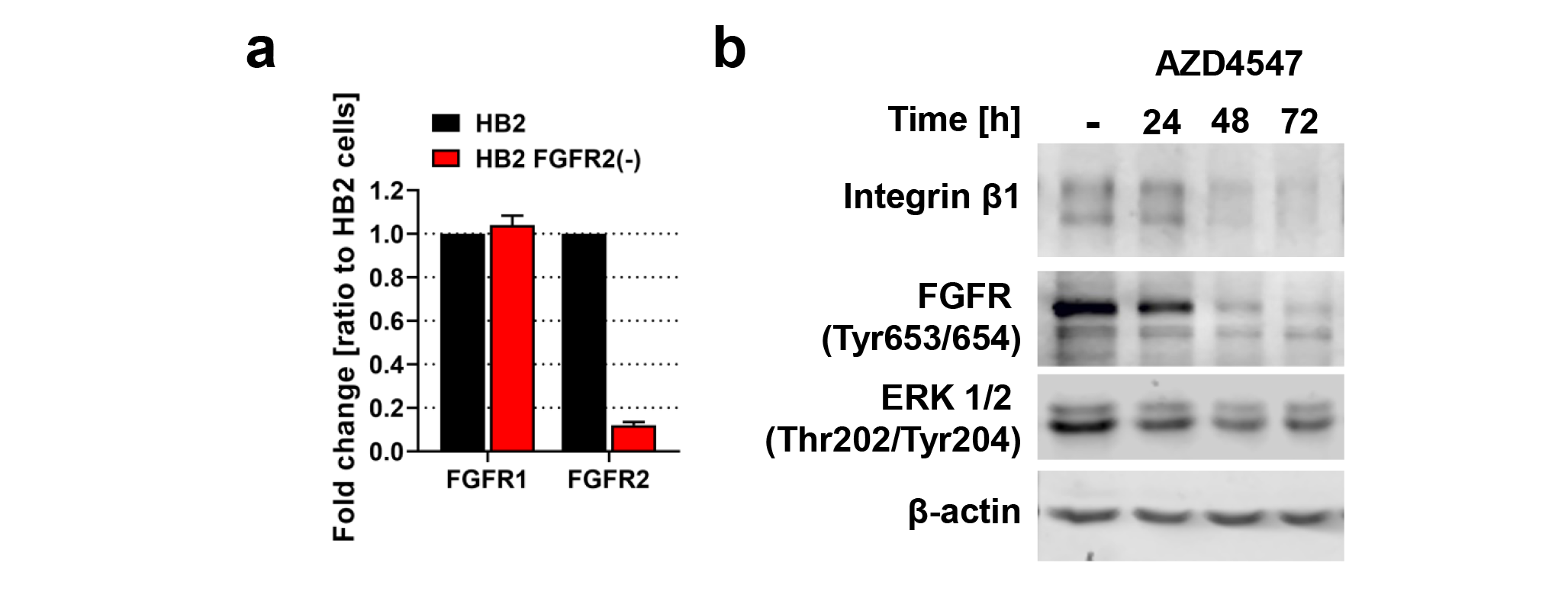

Supplement: Supplementary file 2 — Supplementary Material 2: Fig. S2 (a-b). Densitometry for Western blot analysis of integrin β1 mature form (upper band) degradation pathways in HB2 (a) and HB2 FGFR2(-) (b) cells upon Leupeptin (left panels) and MG132 (right panels) treatment. (c) Localization of LAMP1 and integrin β1 in HB2 (upper panel) and HB2 FGFR2(-) (lower panel) cells. Accumulation of LAMP1 stained lysosomes indicated by arrowheads. Representative pictures taken from at least three independent experiments. Scale bar: 10 μm. [file 10911_2023_9537_MOESM2_ESM.tif]
